# Meta-Analysis of Gene Expression Changes in the Blood of Patients with Mild Cognitive Impairment and Alzheimer’s Disease Dementia

**DOI:** 10.3390/ijms20215403

**Published:** 2019-10-30

**Authors:** Virginie Bottero, Judith A. Potashkin

**Affiliations:** Center for Neurodegenerative Disease and Therapeutics, The Chicago Medical School, Rosalind Franklin University of Medicine and Science, North Chicago, IL 60064, USA; virginie.bottero@rosalindfranklin.edu

**Keywords:** mild cognitive impairment, Alzheimer’s disease, dementia, gene expression, network analysis

## Abstract

Background: Dementia is a major public health concern affecting approximately 47 million people worldwide. Mild cognitive impairment (MCI) is one form of dementia that affects an individual’s memory with or without affecting their daily life. Alzheimer’s disease dementia (ADD) is a more severe form of dementia that usually affects elderly individuals. It remains unclear whether MCI is a distinct disorder from or an early stage of ADD. Methods: Gene expression data from blood were analyzed to identify potential biomarkers that may be useful for distinguishing between these two forms of dementia. Results: A meta-analysis revealed 91 genes dysregulated in individuals with MCI and 387 genes dysregulated in ADD. Pathway analysis identified seven pathways shared between MCI and ADD and nine ADD-specific pathways. Fifteen transcription factors were associated with MCI and ADD, whereas seven transcription factors were specific for ADD. Mir-335-5p was specific for ADD, suggesting that it may be useful as a biomarker. Diseases that are associated with MCI and ADD included developmental delays, cognition impairment, and movement disorders. Conclusion: These results provide a better molecular understanding of peripheral changes that occur in MCI and ADD patients and may be useful in the identification of diagnostic and prognostic biomarkers.

## 1. Introduction

Dementia is a growing public health concern with rising prevalence rates estimated to increase to 131 million worldwide within the next three decades [1]. Cognitive decline is often seen in elderly individuals and in patients who are diagnosed with vascular dementia, Lewy body dementia, frontotemporal dementia, Huntington’s disease, Creutzfeldt-Jakob disease, Parkinson’s disease, and Alzheimer’s disease dementia (ADD). The Alzheimer’s Association estimates that ADD accounts for 60 to 80% of dementia cases and is the sixth-leading cause of death in the United States.

ADD is a progressive disease where dementia gradually worsens over a number of years. The diagnostic criteria for ADD have evolved as we have gained a better understanding of the disease. Before 1984, criteria were focused on the advanced stage of the disease and were based solely on cognitive decline. Since 2011, AD has been identified as a clinical-biological framework based on the National Institute on Aging–Alzheimer’s Association (NIA–AA) criteria and now, the utilization of biomarkers for in vivo detection of AD pathophysiology has been recommended for clinical trials and medical practice [2]. The preclinical phase may start as early as 20 years before the appearance of symptoms. During this period, modifications in the brain such as amyloid buildup and cellular damages are observed. In early ADD stages, patients suffer from short-term memory loss but may also present with other cognitive impairment such as the ability to find words or make judgments. Mild cognitive impairment (MCI) patients are characterized by memory and/or other cognitive problems that are greater than normal for a person’s age and education, but do not interfere with his or her independence. A MCI patient may or may not progress to ADD, but is at significantly increased risk for conversion. At the clinical stage, ADD patients present with cognitive impairment, memory loss, word-finding difficulties, and visual/spatial deficiencies that affect their quality of life and result in the need for assisted living and the inability to work. A recent study determined that the addition of a biological diagnostic to the clinical diagnostic is a more powerful method because it includes ADD asymptomatic patients [3].

The brains of ADD patients are characterized by the accumulation of amyloid-beta (Aβ) plaques and hyperphosphorylated tau in the form of neurofibrillary tangles. Familial ADD patients develop early onset ADD and have mutations in the amyloid precursor protein (*APP*), presenilin 1 (*PSEN1*), and presenilin 2 (*PSEN2*) genes that regulate the production of the Aβ proteins. However, the vast majority of ADD cases present as late onset, usually after age 65, and are considered sporadic. Genetic and environmental factors play a role in the development of sporadic ADD. The epsilon 4 allele of apolipoprotein E (*APOE*) is the greatest genetic factor identified so far that increases the risk of an individual for ADD, possibly accounting for 50% of sporadic ADD patients [4]. The effects of ApoE on cognition are mediated through the classic pathological hallmarks of ADD, Aβ, and Tau [5,6]. In addition to ApoE4, there are over 25 additional genetic risk modifiers for late onset ADD [7,8,9].

The identification of early markers of ADD is critical to diagnosis and therapy. Multiple phase III clinical trials were unsuccessful at treating ADD [10]. This has led to the recognition for the need to intervene at much earlier stages in the disease so as to prevent the cascade of multiple pathologic pathways associated with AD [11]. Imaging techniques and cerebrospinal (CSF) biomarkers have been developed in the last few decades to improve ADD diagnostics. During the preclinical stage of the disease, the National Institute on Aging (NIA) and the Alzheimer’s Association favored the use of amyloid biomarkers [12]. It has been shown that Aβ42 accumulation in the CSF or by PET amyloid imaging of the brain were one of the first abnormalities identified [13]. Whereas CSF and imaging biomarkers are proven to be very efficient, blood biomarkers potentially have important advantages because they would be minimally invasive and relatively inexpensive. Many neural pathologies including ADD are reflected in changes in gene expression, splicing, and protein profiles in brain and CSF, but also in blood, providing a precedent for searching this bodily fluid for biomarkers [14,15,16,17,18].

Microarray and high-throughput technologies for gene expression are essential tools for identifying differential patterns of gene expression that are characteristic of disease. Nonetheless, the interpretation of gene expression patterns can be difficult. Network analysis of gene expression data has identified disease-causing genes, protein biomarkers, and biological pathways associated with ADD [19]. Network analysis of eight genes associated with ADD identified 31 new candidate genes related to late-onset ADD [20]. Additional network analyses using gene expression data from postmortem ADD patients’ brain tissue identified molecular networks that are relevant to ADD pathology [21,22,23,24]. Network analysis was also used to identify genes and miRNAs that correlate with different stages of the disease progression [25,26,27].

Integrative approaches combining multiple data sources have been widely used to identify susceptibility genes that are involved in ADD pathogenesis [28,29,30]. Meta-analysis of microarray datasets determined ADD-specific genes signatures that are specific to the brain [31,32,33,34,35,36]. In addition, the meta-analysis of expression data from the blood of ADD patients identified several miRNAs as promising biomarkers for ADD [37]. By comparing the brain transcriptome of ADD patients to that of patients with other neurodegenerative diseases, seven genes were identified that are ADD-specific [38]. By comparing ADD and Parkinson’s disease transcriptomes, it has become clear that these two diseases share some dysregulated gene expressions patterns [39]. In addition, ADD and ischemic stroke share transcriptionally dysregulated genes [40]. Finally, systems biology approaches identified common pathophysiological mechanisms that are shared between ADD and type 2 diabetes [41,42].

While research on the progression of ADD thus far has been productive, a better understanding of the affected genes and pathways that are involved in the pathogenesis of ADD is needed before we can accurately and reliably detect the pre-clinical stages of the disease as well as predict risk. In the present study, we performed transcriptomic and network analyses of gene expression datasets from MCI as well as early and late ADD patients to better understand the shared molecular pathways that are dysregulated in both forms of dementia and to shed more light on how ADD progresses.

## 2. Results

### 2.1. MCI and ADD Gene Expression Meta-Analysis

**Figure 1:**
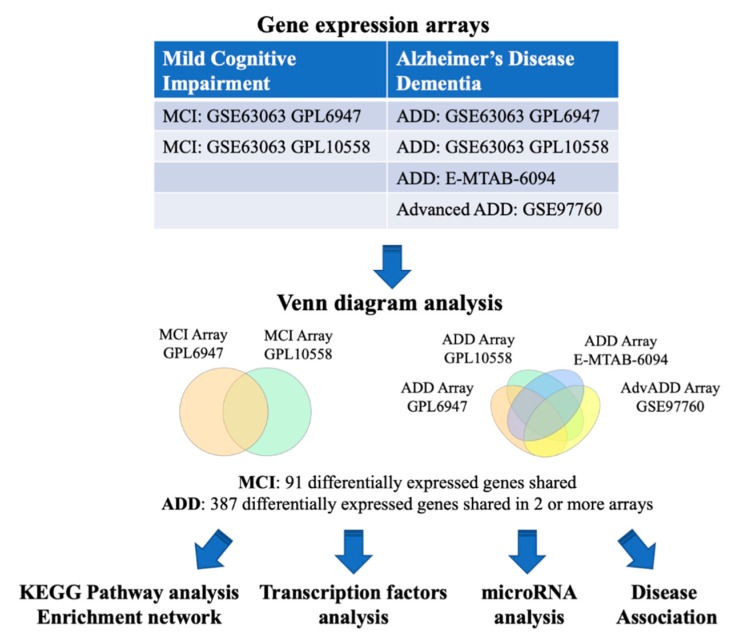
Flowchart of the study. The BaseSpace Correlation Engine (BSCE) was searched and blood gene expression studies with data from mild cognitive impairment (MCI) and Alzheimer’s disease dementia (ADD) were included in this study. Venn diagram analysis was used to identify shared dysregulated genes. The MCI and ADD dysregulated genes were analyzed for shared functional pathways, transcription factors, and miRNAs regulation as well as disease associations. The arrows represent the flow of the steps in the study.

**Figure 2:**
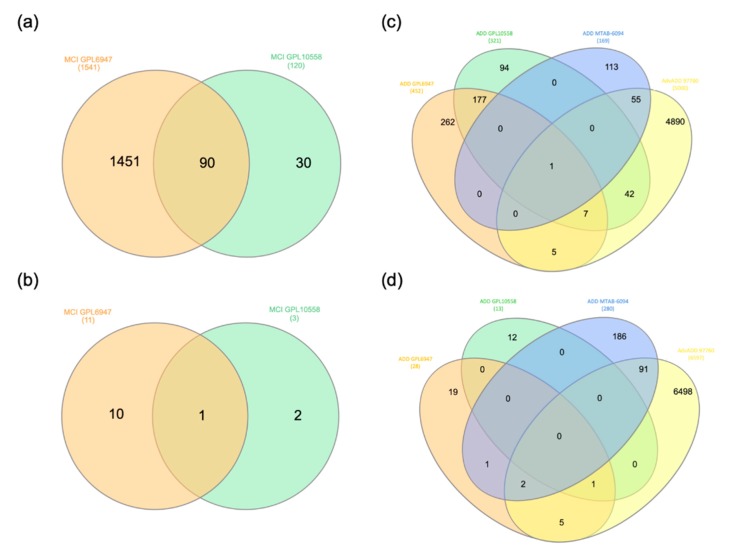
Venn diagram analysis of the genes down and up regulated in MCI and ADD. (**a**,**b**) The genes downregulated (**a**) and upregulated (**b**) in the MCI arrays (GSE63063, GPL10558, and GPL6947 platforms) were downloaded from BSCE and analyzed by Venn diagrams using the following website http://www.interactivenn.net/. (**c**,**d**) The genes downregulated (**c**) and upregulated (**d**) in the ADD arrays (GSE63063 GPL10558 and GPL6947 platforms, MTAB-6094, and GSE97760) were downloaded from BSCE and analyzed by Venn diagrams.

We first identified common genes that are dysregulated in MCI and ADD (Table 1). The overall analysis strategy is presented in Figure 1. The two microarray datasets that were obtained from the BaseSpace Correlation Engine (BSCE) from blood tissue of MCI patients were analyzed by Venn diagram (Figure 2a,b, Tables S1 and S2) and UpSetR analysis (Figure S1). From this, 91 differentially expressed genes were identified in MCI. Similarly, the analysis of the four microarray datasets from blood tissue of ADD patients identified 387 genes that were shared in at least 2 out of the 4 arrays (Figure 2c,d and Figure S1, Table S2). Out of the 387 ADD genes, 10 of them were shared in 3 arrays (*AIF1*, *C9orf78*, *CMTM2*, *GNG11*, *PPBP*, *PPP2R5C*, *RGS10*, *HELZ2*, *SNTB2*, *RNA28S5*) and only *SYF2* was shared in all 4 studies. Finally, it is interesting to note that 85 of the MCI genes are also dysregulated in ADD (Table S3).

### 2.2. Pathway Enrichment Analysis

**Figure 3:**
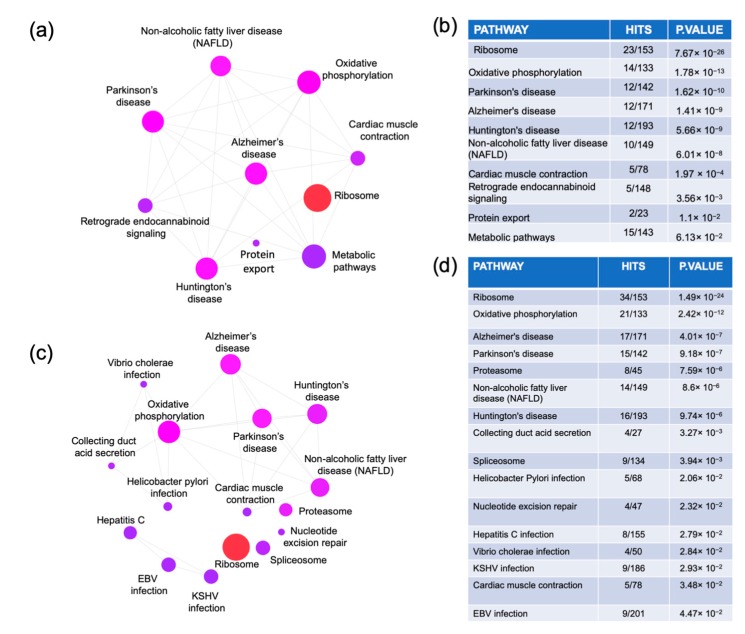
Pathway analysis. The genes that were commonly dysregulated in the MCI arrays (**a**,**b**) and in at least two of the ADD arrays (**c**,**d**) were obtained from the Venn diagram analysis. The genes lists were uploaded to https://www.networkanalyst.ca/NetworkAnalyst/faces/home.xhtml where an enrichment network analysis was performed using the Kyoto Encyclopedia of Genes and Genome (KEGG) database.

The MCI and ADD dysregulated genes were uploaded to NetworkAnalyst to perform a pathway enrichment network analysis using the Kyoto Encyclopedia of Genes and Genome (KEGG) database (Figure 3). The MCI analysis identified 10 pathways: ribosome, oxidative phosphorylation, Parkinson’s disease, Alzheimer’s disease, Huntington’s disease, non-alcoholic fatty liver disease, cardiac muscle contraction, retrograde endocannabinoid signaling, and protein export and metabolic pathways. The ADD analysis revealed 15 pathways. Seven pathways were common with MCI analysis (ribosome, oxidative phosphorylation, Parkinson’s disease, Alzheimer’s disease, Huntington’s disease, non-alcoholic fatty liver disease, and cardiac muscle contraction). Nine pathways were ADD-specific (proteasome, collecting duct acid secretion, spliceosome, *Helicobacter pylori* infection, nucleotide excision repair, hepatitis C infection, *Vibrio cholerae* infection, Kaposi’s sarcoma-associated herpesvirus (KSHV) infection, and Epstein–Barr virus (EBV) infection). The list of genes corresponding to each of these pathways is presented in Table S4.

### 2.3. Gene-Transcription Factors Interaction Analysis

**Figure 4:**
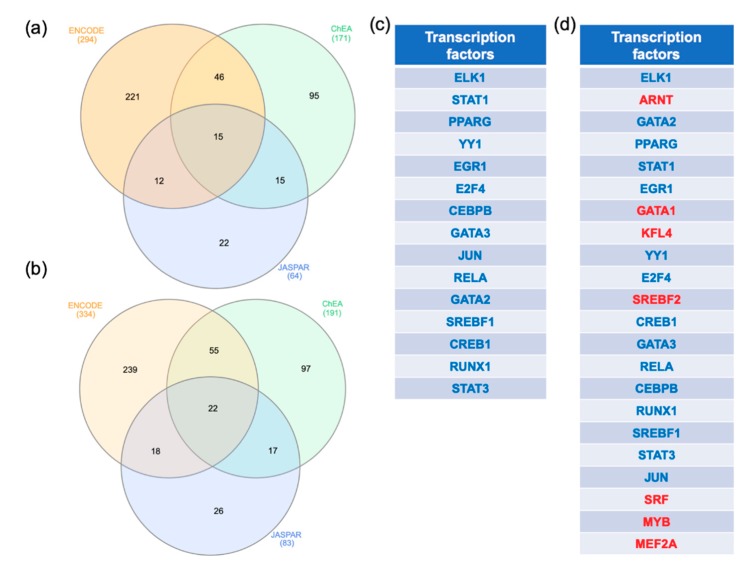
Transcription factors analysis. The genes commonly dysregulated in the MCI arrays and in at least two out of the ADD arrays were obtained using Venn diagram analysis. The gene lists were uploaded to https://www.networkanalyst.ca/NetworkAnalyst/faces/home.xhtml. The gene-transcription factor interaction network was performed with ENCODE, ChEA, and JASPAR. A Venn diagram analysis was performed to identify the transcription factors identified by the three methods. (**a**,**b**) represent the results of the Venn diagram analysis performed with MCI and ADD genes, respectively. The transcription factors interacting with the MCI and ADD genes were listed in (**c**,**d**), respectively. Transcription factors in blue are common in MCI and ADD analysis whereas the transcription factors in red were specific to ADD regulation.

In order to identify key regulators of the MCI and ADD dysregulated genes, a transcription factor analysis was performed. The gene transcription factor interactomes were performed on NetworkAnalyst using three different databases (ENCODE, ChEA, and JASPAR). The transcription factors that were shared by all the databases were identified by Venn diagram analysis (Figure 4a,b). In the MCI analysis, 15 transcription factors (Figure 4a,c) and in the ADD analysis, 22 transcription factors, respectively, were shared by all the databases (Figure 4b,d). Interestingly, all the transcription factors regulating the MCI genes are shared with ADD (ELK, STAT1, PPARG, YY1, EGR1, E2F4, CEBPB, GATA3, JUN, RELA, GATA2, SREBF1, CREB1, RUNX1, STAT3). Seven transcription factors were specific to the ADD genes (ARNT, GATA1, KFL4, SREBF2, SRF, MYB, MEF2A).

### 2.4. Gene-miRNA Interaction Analysis

To further understand the regulation of MCI and ADD genes, a gene-miRNA interaction network analysis was performed in NetworkAnalyst. Comprehensive experimentally validated miRNA-gene interaction data was collected from TarBase and miRTarBase. The MCI genes resulted in the identification of three subnetworks. The first subnetwork had 58 genes as seeds and resulted in 686 miRNAs. The second subnetwork was centered on *UQCRH*, which can be regulated by 16 miRNAs. Finally, the third subnetwork was centered on *COX7A2*, which can be regulated by 2 miRNAs. Altogether, 704 miRNAs were identified as regulators of the MCI genes. These miRNAs were ranked by decreasing degree followed by decreasing betweenness. The top MCI miRNAs, with a degree superior or equal to 5, were listed in Table 2. The ADD genes network identified 1773 miRNAs that were ranked based on their degrees and betweenness. The top miRNAs (degree superior or equal to 20) are listed in Table 2. A Venn diagram analysis was performed to determine the shared miRNAs that are involved in MCI and ADD regulation and 683 miRNAs were identified. Among the highly ranked miRNAs listed in Table 2, all the miRNAs that may regulate the MCI genes are shared with the ADD genes, except Hsa-mir-335-5p, which is unique to the ADD genes and may regulate genes that are involved in late stages of ADD.

### 2.5. Gene-Disease Association Analysis

A gene-disease association network analysis was performed in NetworkAnalyst. The MCI genes resulted in the identification of three subnetworks. Altogether, 119 associated diseases were identified with the MCI genes. These associated diseases were ranked by decreasing degree followed by decreasing betweenness. The top MCI associated diseases, with a degree superior or equal to 3, are listed in Table 3. The ADD genes network identified 631 associated diseases that were ranked based on their degrees and betweenness. The top associated diseases (degree superior or equal to 6) are listed in Table 3. The full list of diseases that are associated with MCI and ADD are listed in Table S5. Interestingly, most of the diseases that are associated with the MCI and ADD genes are connected to developmental delay, cognition impairment, and movement disorders. A Venn diagram analysis was performed to determine the shared associated diseases between MCI and ADD. All the associated diseases that were unveiled with MCI genes were also associated with the ADD genes (Table S5).

## 3. Discussion

### 3.1. MCI and ADD Dysregulated Genes

ADD has a long preclinical phase where the disease may start as early as 20 years before the appearance of symptoms. During the preclinical phase in ADD, mild cognitive impairment is common. By meta-analysis of MCI and ADD gene expression arrays studies, we identified dysregulated peripheral pathways in the blood of MCI and ADD patients that are potentially useful for identifying biomarkers.

The transcriptomic analysis of two MCI studies identified 91 dysregulated genes. Several of them have previously been linked to ADD. The levels of diazepam-binding inhibitor (*DBI*) were downregulated in both blood arrays. The levels of DBI in the cerebrospinal fluids were elevated in parkinsonian subjects with dementia and in patients with ADD [47]. DBI was decreased in ADD hippocampal protein extracts, whereas RNA sequencing of the parietal cortex of ADD patients suggested an upregulation of *DBI* [48,49]. The dysregulated MCI genes included those coding for the NADH: Ubiquinone Oxidoreductase subunits (*NDUFA1*, *NDUFB2*, *NDUFA4*, *NDUFB3*, and *NDUFB3*), all of which were downregulated in the MCI arrays. Interestingly, *NDUFA1* and *NDUFB2* were identified as central genes in a functional motif network using hippocampal and cell model transcriptomic data [50]. NDUFB3 was decreased in the brain proteome of late onset ADD patients [51] and its gene expression was decreased in the entorhinal cortex of ADD patients stages V to VI [52]. In addition, NDUFA4 was also down-regulated in the brain proteome of early onset ADD individuals [51].

Of interest, 37 ribosomal subunit genes were downregulated in MCI. Earlier studies showed that several ribosomal subunits are reduced in ADD. RPL26 staining was reduced in neurons of layer II of the entorhinal cortex in ADD patients [53]. RPL35A was reduced in the retina of aged APP/PS1 (ADD-like) mice [54]. By comparing blood and brain transcriptomes in a systematic analysis, an RNA signature comprising *NDUFA1*, *MRPL51*, and *RPL36AL* discriminated between ADD patients and controls with high sensitivity and specificity [55]. A prominent gene in the regulation of inflammation, *S100A8*, is downregulated in the MCI blood arrays that were analyzed. According to the gene expression results, S100A8 protein levels were decreased in the serum of ADD patients [56]. Interestingly, S100A8 also delays Aβ aggregation, suggesting a regulatory function [57]. Finally, the levels of thioredoxin-1 (*TXN*), an anti-oxidant, were reduced in the blood of MCI patients in the RNA arrays that were analyzed. In accordance with this result, a decrease in the protein level of TXN was also observed in the hippocampus and cerebellum of amnesic MCI patients [58]. TXN protein is decreased in the amygdala, superior and middle temporal gyri, and hippocampus/parahippocampal gyrus of ADD patients [59]. In addition, altered cellular localization or oxidized forms of TXN may affect its function in ADD patients [60,61].

The blood transcriptomic meta-analysis of ADD patients identified *SYF2* as the only mRNA that was downregulated in all four studies. This pre-mRNA splicing factor is involved in cell cycle progression and may be involved in neuroinflammation [62]. The transcriptional level of the regulator of G-protein signaling 10 (*RGS10*) was downregulated in three out of the four studies that were analyzed. It has been proposed that RGS10 may have a protective role in neuronal neuroinflammation [63]. The mRNA levels of the platelet basic protein *PPBP* were downregulated in two studies. Interestingly, a downregulation of PPBP protein levels was also detected in the serums of ADD patients compared with healthy controls [56].

### 3.2. MCI and ADD Enrichment Pathways

Network and pathway analysis revealed that all the pathways that were identified from the analysis of the MCI genes are shared with ADD, strongly suggesting that MCI often progresses to ADD. The most highly ranked pathway was the ribosomal pathway. Similar to this finding, the ribosomal pathway was also enriched in a network analysis that was performed using other datasets (GSE4226 and GSE4229) [64]. The ribosomal pathway was also significantly enriched in the differentially expressed genes from the neuronal dataset GSE4757 [65]. In addition, it has been shown that the transcriptional level of several ribosomal proteins genes were altered in the hippocampus of ADD patients and an impairment in protein synthesis was observed in the hippocampus and the superior middle temporal gyrus of MCI and ADD patients [66,67]. Finally, pathogenic Tau interacts closely with ribosomes in ADD, resulting in a reduction of protein synthesis, affecting synapses, and eventually leading to cognitive impairment [68].

The oxidative phosphorylation pathway was the second most highly ranked dysregulated pathway in both MCI and ADD. Recently, this pathway was enriched in ApoE4 hippocampal proteomic analysis [69]. Mitochondrial dysfunction and oxidative damage have been shown to have a crucial role in the pathogenesis of ADD [70]. It has been proposed that mitochondrial dysfunction is present in MCI patients and eventually leads to ADD [71,72].

Another pathway that is shared between MCI and ADD is the non-alcoholic fatty liver disease pathway (NAFLD). Interestingly, NAFLD was also enriched in a proteomic study from hippocampal extracts from APP/PS1 mice [69]. It has been shown that the liver contributes to the clearance of circulating Aβ in the periphery [73]. Clinical studies have demonstrated an association between NAFLD and cognitive impairment in adults [74]. In addition, it has been shown that the content of n-3 polyunsaturated fatty acids in the liver strongly correlates with the cognitive status in patients with ADD [75]. The levels of docosahexaenoic acid, a neuroprotective fatty acid, are reduced in the brain and liver of ADD patients and correlate with cognitive impairment [75].

The cardiac muscle contraction pathway was also shared between MCI and ADD. An association between ADD and cardiovascular diseases has been observed. Several genes mutated in ADD have been implicated in heart dysfunction. For example, mutation in presenilin 1 (*PSEN1*) and presenilin 2 (*PSEN2*) are associated with cardiomyopathies [76]. Interestingly, in an ADD mouse model containing *APP* and *PSEN1* mutations, cardiomyocytes presented with mechanical dysfunction [77]. The cognitive function of ADD patients with cerebrovascular atherosclerosis declined faster compared to ADD patients without cerebrovascular atherosclerosis [78].

Interestingly, some dysregulated pathways were specific to ADD patients. The role of the proteasome system in the development of ADD has been highly documented (reviewed in [79]). A two-way relationship between Aβ and the proteasome has been observed. Proteasome activity decreases in brain areas that are affected by ADD [80]. This decrease of proteasome activity seems to be dependent on Aβ accumulation [81,82,83]. In addition, it has been proposed that the proteasome system degrades Aβ and decreases its toxicity, indicating that a decrease of proteasome activity might consequently be responsible for an increase of Aβ [84]. Our meta-analysis indicates that proteasome activity might also be reduced due to a decrease in the transcriptional level of different proteasomal subunits genes.

A second affected pathway that is specific to ADD compared to MCI patients is the spliceosome. The re-localization and aggregation of the spliceosome protein U1 snRNP, a small nuclear ribonucleoprotein, has been observed in ADD pathology [85,86,87,88]. The accumulation of U1 snRNP resulted in changes in RNA splicing [89,90]. Aberrant splicing in presenilin 2 is present in sporadic ADD patients [91]. Furthermore, altered splicing of the receptor for advanced glycation end products (RAGE) occurs in ADD [92]. In our analysis, we observed that the genes that are involved in the spliceosome were downregulated in ADD. This result is in accordance with a proteomic study in which a downregulation of hnRNP-related proteins was observed after Aβ peptide incubation on neuroblastoma cells [93]. More recently, a pathway enrichment analysis of the hippocampal proteome from ApoE4 mice compared to that of wild type highlighted the importance of the spliceosome in ADD [69].

Viral and bacterial infection pathways are also dysregulated in ADD. Several studies indicated a link between viral infection, the development of cognitive impairment, and ADD [94,95,96,97,98]. In addition, *H. pylori* infection has been associated with a reduction of cognition and ADD [99,100,101]. Furthermore, *H. pylori*-specific IgG antibody levels are significantly increased in blood and CSF of ADD patients [102]. At the molecular level, it has been shown that *H. pylori* infections induced an increase of tau phosphorylation [103].

Finally, the nucleotide excision repair pathways were identified as being dysregulated in ADD. The DNA damage that is caused by oxidative stress is usually repaired by nucleotide excision repair, a mechanism in which a damaged region of DNA is cut out and replaced by DNA synthesized using the undamaged strand as a template. The presence of oxidative stress in ADD is well established and most likely induces DNA damage [104]. The expression of several nucleotide excision repair components was altered in blood and postmortem brain tissue in MCI and ADD patients [105]. It has been shown that blood mRNA levels of 8-Oxoguanine DNA Glycosylase (*OGG1*), an enzyme that is responsible for the excision of mutated 8-Oxoguanine, was low in MCI and ADD. In addition, the blood mRNA level of Poly(ADP-Ribose) Polymerase 1 (*PARP1*), an enzyme that is involved in DNA repair enzyme by inducing the poly-ADP-ribosylation of proteins, was high in MCI and ADD. In the dataset that was analyzed, we observed a decreased expression of nucleotide excision repair genes in MCI and ADD, whereas advanced ADD datasets indicated increased expression, suggesting that these genes might be involved in early ADD events.

Altogether, this study suggests the existence of shared molecular pathways between MCI, ADD, Parkinson’s disease, and Huntington’s disease. In addition, the importance of mitochondrial function, ribosome, proteasome, and inflammation were revealed. Further research is expected to determine the roles of additional pathways including non-alcoholic fatty liver disease and nucleotide excision repair.

### 3.3. Transcription Factors that May Regulate MCI and ADD Genes Expression in Blood

We performed a gene-transcription factor interactome network to identify the transcription factors that may regulate the genes in blood in MCI and ADD. Fifteen transcription factors were enriched in MCI and ADD including ELK, STAT1, PPARG, YY1, EGR1, E2F4, CEBPB, GATA3, JUN, RELA, GATA2, SREBF1, CREB1, RUNX1, and STAT3. In accordance with our study, a network analysis from the array GSE4757 identified several transcription factors implicated in the pathogenesis of ADD [65]. Indeed, similar to our result, GATA2, YY1, PPARPG, and JUN were among the key regulatory molecules associated with transcriptional changes for ADD. More recently, the analysis of two other ADD microarray datasets (GSE4226 and GSE4229) also proposed a role for GATA2, PPARG, and YY1 as transcriptional regulators [64]. Yin Yang 1 (YY1) is implicated in neurodegenerative diseases [106,107,108]. This transcription factor is dysregulated in ADD brains and may play a role in the regulation of BACE1 and APP expression [109,110,111,112]. The analysis of hippocampal gene expression in ADD patients showed the deregulation of STAT3 and CEBPB [113].

In our analysis, seven transcription factors were specific to ADD including ARNT, GATA1, KFL4, SREBF2, SRF, MYB, and MEF2A. GATA-1 may regulate APP gene expression, whereas the transcription factor SREBF2 may be involved in the regulation of BACE1 expression [114,115]. In addition, APP/PS1 mice overexpressing SREBP-2 exhibit combined Aβ accumulation and tau pathology [116]. Interestingly, it has been suggested that the expression levels of SREBP-2 could influence the survival rate of late onset ADD patients [117]. The transcription factor SRF was identified as a key regulator in ADD pathology [65]. The transcription factors MYB and MEF2A were respectively involved in protection for Aβ toxicity and in neuronal survival [118,119].

### 3.4. miRNAs that May Regulate MCI and ADD Gene Expression in Blood

To identify the microRNAs that may regulate the MCI and ADD genes, we performed a gene-microRNA interactome network. We ranked the microRNA based on the degree and betweenness in these networks. Whereas the analysis identified numerous potential microRNAs, we focused on the top ranked MCI and ADD microRNAs. All the microRNAs that were identified as potentially regulating MCI genes were also identified for ADD genes, confirming the close relationship between the two diseases and suggesting that they might be involved in early development of the pathology. Previous studies suggest that several of these miRNAs play a role in dementia and neurodegeneration. A meta-analysis study showed that mir-16-5p may regulate gene expression in ADD [64]. Mir-16-5p is differentially expressed in the CSF-derived exosomes of early onset ADD patients and in the brains of late onset ADD patients [120,121]. The upregulation of mir-92a-3p was observed in the plasma of both MCI and ADD subjects [122]. Mir-26b-5p expression is altered by Aβ_1–42_ [123]. Mir-106b-5p expression in the blood was proposed as an ADD biomarker and predicted ADD with 93% specificity and 68% sensitivity [124]. Let-7a-5p is proposed as an ADD blood biomarker and its expression is downregulated [125]. MiRNAs belonging to the mir-20a family, such as mir-17-5p, could regulate APP expression [126]. Mir-17 is upregulated in the serum of patients with multiple system atrophy [127]. Mir-155 is triplicated in Down syndrome [128,129]. It has been proposed that mir-155 upregulation is correlated with Down syndrome dementia [130,131]. Mir-155 plays a role in regulating the memory impairment in ADD rats [132]. It has been proposed that mir-155 contributes to ADD neuroinflammation [133,134]. Mir-155-5p and mir-26b-5p expression is altered in ADD mouse models and in patients [135,136]. Interestingly, mir-155-5p is also upregulated in the blood of PD patients, indicating that this miRNA might have a general role in neurodegeneration [137]. Similar to our study, a network analysis identified mir-20a, mir-17, mir-155, mir-18, and mir-106b as potential regulators of the expression genes that are altered in an ADD mouse model [138]. Only one miRNA, mir-335-5p, was specific to ADD, suggesting that it may be useful as an ADD biomarker.

### 3.5. Limitations

As in all microarray and meta-analyses, our study has some limitations. The sample sizes of each microarray study will affect the power of our analysis. Regarding this, we have selected only the microarray data sets that have five or more patients or controls for our meta-analysis. Analysis of additional datasets when they become available is expected to provide information that will be useful for determining if our results may be replicated. The microarray data sets that were used in this study are heterogeneous because they came from multiple studies using different conditions and number of samples. In order to minimize the effect of this heterogeneity, we only used studies that were curated by the BSCE database, which controls for the quality and normalization of the data. Genes of which their mean normalized test and control intensities were both less than the 20^th^ percentile of the combined normalized signal intensities were removed.

Our analyses were performed based on publicly available data. The site-to-site variability may influence the identification of dysregulated genes. Whereas the methodology of blood collection and RNA preparation is often similar, the diagnostic criteria might be variable between the different studies performed in the USA, Europe, and Brazil. For example, with a deeper understanding of the disease, the National Institute of Aging (NIA) in the USA has revised the clinical diagnostic guidelines several times. For research purposes, it has been proposed to define the research cohort based on biological criteria. The use of cognitive tests to identify the healthy population instead of biological tests might have been the best approach at the time the original study was done. It is possible, therefore, that some healthy controls might be preclinical MCI or ADD patients. In this regard, it has been estimated that 30–40% of cognitively normal individuals have some sign of neuropathological brain changes at autopsy [139].

Another limitation of this study is that patient information in general was incomplete and often restricted to age. Other factors, such as genetic determinants, comorbidities, and an individual’s lifestyle choices, most likely influence gene expression, but this information was not available. For example, the presence of ApoE4, the most common risk factor of ADD, is often missing from the patients’ profiles. ADD is strongly associated with vascular disease or vascular risk factors [140]. In addition, mid-life elevated blood pressure may increase the risk of ADD later in life [141]. Another well documented risk to develop ADD is diabetes mellitus [142]. Impaired glucose tolerance and ADD have been correlated in different patient studies [143,144,145]. Finally, an association has also been observed between ADD and low bone mineral density or osteoporosis [146]. Information such as blood pressure, glucose levels, thyroid hormone levels, bone mineral density, and comorbidities would be interesting to collect in future studies in order to determine if they affect gene expression in the blood.

Other information that was unfortunately missing from the patients was their concomitant medications. We compared the MCI and ADD genes that were identified in this study to the genes that were targeted by the approved or investigational ADD drugs [147]. *S100P*, which is present in both MCI and ADD, could be targeted by the investigational drug ALZT-OP1a/b. Among the ADD specific-gene, *CA1* could be targeted by calcium channel blockers, whereas *SNCA* could be targeted by Resveratrol. The patients in the ADD cohorts may be receiving different medications to alleviate their symptoms such as BDZ, barbiturates, mood stabilizers (valproate, lamotrigine, etc), SSRI, NNRI, mixed effect compounds, and neuroleptics. In addition, they could also be taking drugs for other conditions. All these drugs have the potential to dysregulate gene expression and potentially alter the results of this study. However, it should be noted that many genes that found to be altered in MCI were also altered in early ADD, suggesting a common mechanism and relevance to disease pathogenesis. Further studies should incorporate the patients’ medications in the characteristics.

ADD is now considered a continuum rather than a disease and is divided into three distinct stages of presymptomatic, MCI, and ADD. Further analysis could include correlation analysis of key gene expression data with biological or cognitive results. Longitudinal cohort studies will be necessary to better understand the correlation between potential RNA biomarkers and the signs or symptoms of the disease.

## 4. Materials and Methods

### 4.1. Analysis of Blood Transcriptomic Studies

We used the curated database BaseSpace Correlation Engine (BSCE, Illumina, Inc., San Diego, CA, USA) to search for gene expression studies in MCI, ADD, and advanced ADD [148]. Using the search terms “Alzheimer’s disease”, “mild cognitive impairment”, “blood”, “human”, “RNA”, and “microarray”, we identified three studies with blood from MCI and ADD patients. Only human microarray studies with five samples or more for cases and controls and that were curated in BSCE were considered for analysis. Two studies on ADD patients were removed because they contained less than five samples. Six microarrays met our inclusion criteria as of July 1, 2019. A description of microarray datasets that were included in this study is provided in Table 1.

The diagnosis of ADD subjects in GSE63063 was performed according to guidelines from the National Institute of Neurological and Communicative Disease and Stroke and Alzheimer’s disease (NINCDS-ADRDA) and Diagnostic and Statistical Manual of Mental Disorders (DSM-IV) [43,44,45]. Subjects with MCI reported problems with memory, corroborated by an informant, but had normal activities of daily living as specified in the Petersen’s criteria for amnestic MCI. MCI subjects scored 0.5 on the total Clinical Dementia Rating Scale or had a memory score of 0.5 to 1. All subjects underwent a structured interview and a battery of neuropsychological assessments including the Mini Mental State Examination (MMSE), Global Deterioration Scale (GDS), and Clinical Dementia Rating Scale (CDR) by trained researchers. Healthy controls and MCI subjects were further assessed using the Consortium to Establish a Registry for Alzheimer’s Disease (CERAD) battery. The MCI cohort was composed largely of subjects with a likely ADD-endpoint. Subjects were excluded from the study if they were younger than 65 years, had significant neurological or psychiatric illness other than ADD, had significant systematic illness or organ failure, or had a geriatric depression rating scale score ≥4/5. More details can be found in the literature [43,44,45]. Subjects in the dataset GSE97760 were all female, including patients with advanced ADD (*n* = 9, age 79.3 ± 12.3 years) and age–matched female healthy controls (*n* = 10, age 72.1 ± 13.1 years) [46]. The ADD diagnoses were made by the Neurobehavior and Memory Disorders Clinic at the Ohio State University Wexner Medical Center (NMDC-OSUWMC), following the revised NIH Diagnostic Guidelines for Alzheimer’s disease and Related Disorders [46]. All recruited ADD subjects were nursing home residents and were completely dependent or bed-ridden, with a severe clinical dementia rating of 2 to 3 at the time of recruitment. Healthy controls were recruited among female spouses and primary caregivers of afflicted male dementia patients seen at MDC-OSUWMC, and were free of dementia, acute or chronic infection, inflammation, and diabetes. More details can be found in [46]. Information about the diagnosis of ADD patients from E-MTAB-6094 is not available. Subjects in the dataset E-MTAB-6094 were 14 female and 8 male patients with ADD (*n* = 22, age 79.4 ± 6.6 years) and 10 female and 3 male age–matched healthy controls (*n* = 13, age 77.3 ± 6.2 years). All ADD patients were diagnosed based on the criteria established by the Diagnostic and Statistical Manual of Mental Disorders 4th edition and the National Institute of Neurological and Communicative Disorders and Stroke and The Alzheimer’s disease Related disorders Association. Due to the lack of neuropathological exams, patients were classified as having probable Alzheimer’s disease.

The differentially expressed genes were curated by BSCE. Statistical analyses were performed on log scale data. In the parametric test, variances were not assumed equal (Welch *t*-test). A *p*-value cutoff of 0.05 and fold change of 1.2 was applied to generate the final list of genes. Genes of which their mean normalized test and control intensities are both less than the 20^th^ percentile of the combined normalized signal intensities were removed. Final gene expression data from microarray studies were downloaded from BSCE (Table S1). A Venn diagram analysis was performed with the genes that were up or downregulated in the two MCI arrays and the four ADD arrays independently. The transcription factors Venn diagram was created using the website http://bioinformatics.psb.ugent.be/webtools/Venn/, whereas the UpSetR diagram was created using the website https://gehlenborglab.shinyapps.io/upsetr/ [149]. Only genes that were differentially expressed in both MCI arrays and in at least two ADD studies were included for further analysis.

### 4.2. Pathway Enrichment Analysis

An official gene symbol from the genes that were identified in the meta-analyses were imported into NetworkAnalyst 3.0 for pathway analyses [150,151]. The Kyoto Encyclopedia of Genes and Genome (KEGG) pathway database was used as annotation sources [152].

### 4.3. Gene-Transcription Factors Interaction Analysis

Gene-transcription factors interactome was performed in NetworkAnalyst. Transcription factor and gene target data were derived from the Encyclopedia of DNA Elements (ENCODE) ChIP-seq data, ChIP Enrichment Analysis (ChEA), or JASPAR database [153,154,155]. ENCODE uses the BETA Minus Algorithm in which only peak intensity signal <500 and the predicted regulatory potential score <1 is used. ChEA transcription factor targets databases that are inferred from integrating literature curated Chip-X data. JASPAR is an open-access database of curated, non-redundant transcription factor (TF)-binding profiles. A Venn diagram analysis was performed with the transcription factors that were identified with each database. Transcription factors were ranked according to network topology measurements including degree and betweenness centrality.

### 4.4. Gene-miRNA Interaction Analysis

The gene-miRNA interactome was performed in NetworkAnalyst. The Gene-miRNA Interactome was carried out from comprehensive experimentally validated miRNA-gene interaction data collected from TarBase and miRTarBase [156,157,158].

### 4.5. Gene-Disease Association Analysis

Gene-disease association analysis was performed in NetworkAnalyst. The literature curated gene-disease association information was collected from the DisGeNET database, a publicly available collections of genes and variants that are associated with human diseases [159].

## 5. Conclusions

Collectively, the results that are presented in this study reveal the characteristics that are shared between MCI and ADD. Most of the genes and pathways are already dysregulated in the early preclinical phase of ADD, suggesting the importance of an earlier diagnostic tool. During MCI, genes that are involved in neurodegeneration, ribosome function, oxidative phosphorylation, non-alcoholic fatty liver disease, and cardiac muscle contraction are dysregulated and most likely influence the early stages of pathology. Other pathways, such as viral and bacterial infection, proteasome function, collecting duct acid secretion, spliceosome function, and nucleotide excision repair were specific to ADD and might play a role in the later stages of disease development. In this study, we also identified putative transcriptional and miRNA regulators of MCI and ADD gene expression. Whereas 15 transcription factors might be involved in both MCI and ADD development, seven transcription factors might be involved later in ADD progression. In addition, mir-335-5p was specific for ADD, suggesting that it may be useful as a biomarker.

## Figures and Tables

**Table 1:** Gene expression datasets used in this study.

Disease	Datasets	Platform	Cases	Controls	References
MCI	GSE63063 (GSE63060, GSE63061)	Illumina GPL6947	80	104	[43,44,45]
MCI	GSE63063 (GSE63060, GSE63061)	Illumina GPL10558	109	136	[43,44,45]
ADD	GSE63063 (GSE63060, GSE63061)	Illumina GPL6947	142	104	[43,44,45]
ADD	GSE63063 (GSE63060, GSE63061)	Illumina GPL10558	139	136	[43,44,45]
ADD	E-MTAB-6094	Agilent G4845A	22	13	Not published
AdvADD	GSE97760	Agilent GPL16699	9	10	[46]

Blood transcriptomics studies selected for the analysis. * GSE63063 dataset are composed of two studies: GSE63060 and GSE63061. MCI: Mild cognitive impairment, ADD: Alzheimer’s disease dementia, AdvADD: Advanced Alzheimer’s disease dementia.

**Table 2:** MicroRNA analysis.

Gene	miRNA	Degree	Betweenness
MCI	hsa-mir-16-5p	15	29305
	hsa-mir-92a-3p	11	13618
	hsa-mir-26b-5p	10	16008
	hsa-mir-106b-5p	7	8335
	hsa-mir-93-5p	7	7370
	hsa-mir-20a-5p	7	7297
	hsa-mir-320a	7	3580
	hsa-let-7a-5p	6	11229
	hsa-mir-484	6	3618
	hsa-mir-615-3p	6	3342
	hsa-mir-18a-3p	5	8997
	hsa-mir-7977	5	6593
	hsa-mir-17-5p	5	4484
	hsa-mir-155-5p	5	4269
	hsa-mir-193b-3p	5	3279
	hsa-mir-450a-1-3p	5	3266
	hsa-mir-887-5p	5	1840
ADD	hsa-mir-16-5p	51	101307
	hsa-mir-26b-5p	48	69947
	hsa-mir-92a-3p	33	53149
	hsa-mir-193b-3p	30	65562
	hsa-mir-17-5p	28	45790
	hsa-mir-93-5p	28	32553
	hsa-mir-106b-5p	28	32245
	hsa-mir-20a-5p	26	27726
	hsa-mir-192-5p	26	22623
	hsa-mir-335-5p	25	40587
	hsa-mir-186-5p	24	40049
	hsa-mir-615-3p	24	26450
	hsa-let-7b-5p	23	44950
	hsa-mir-155-5p	23	33976
	hsa-mir-215-5p	23	16736
	hsa-mir-20b-5p	21	16266
	hsa-mir-519d-3p	21	16266
	hsa-let-7a-5p	20	22235

The genes that are commonly dysregulated in the MCI arrays and in at least two of the ADD arrays were obtained using Venn diagram analysis. The genes lists were uploaded to https://www.networkanalyst.ca/NetworkAnalyst/faces/home.xhtml. The gene-microRNA interactome was created using TarBase and miRTarBase databases. The microRNAs with a degree ≥5 that may regulate the MCI genes are listed, the microRNAs with a degree ≥20 that may regulate the ADD genes are listed. In green is the microRNA that was only identified in late ADD.

**Table 3:** Disease association analysis.

Gene	Disease Associated	Degree	Betweenness
MCI	Autosomal recessive predisposition	5	1795
	Failure to gain weight	3	306
	Pediatric failure to thrive	3	306
	Muscle degeneration	3	296
	Neurogenic muscular atrophy	3	296
	Skeletal muscle atrophy	3	296
	Neurogenic muscle atrophy, especially in the lower limbs	3	296
	Cerebellar ataxia	3	161
	Muscle hypotonia	3	161
	Hyperreflexia	3	161
	Global developmental delay	3	161
	Cognitive delay	3	161
	Mental and motor retardation	3	161
	Hypertrophic cardiomyopathy	3	123
ADD	Autosomal recessive predisposition	11	25695
	Liver cirrhosis experimental	9	7088
	Schizophrenia	8	11805
	Strabismus	7	16263
	Failure to gain weight	6	6147
	Pediatric failure to thrive	6	6147
	Hyperreflexia	6	4072
	Muscle hypotonia	6	3821

The genes that are commonly dysregulated in the MCI arrays and in at least two of the ADD arrays were obtained using Venn diagram analysis. The genes lists were uploaded to https://www.networkanalyst.ca/NetworkAnalyst/faces/home.xhtml. The gene-disease association networks were created using DisGeNet databases. The associated diseases with a degree ≥3 that may regulate the MCI genes are listed, whereas the associated diseases with a degree ≥6 that may regulate the ADD gene are listed.

## Supplementary Materials

The following are available online at https://www.mdpi.com/1422-0067/20/21/5403/s1, Table S1. Genes differentially expressed in each arrays. Table S2. Shared genes between the MCI arrays and the ADD arrays. Table S3. Specific and shared genes from the MCI and ADD meta-analysis. Table S4. Genes in each pathway. Table S5. Associated disease. Figure S1: UpsetR analysis of the genes down and up regulated in MCI (A and B, respectively) and of the genes down and up regulated in ADD (C and D, respectively). The UpSetR diagram was created using https://gehlenborglab.shinyapps.io/upsetr/.
