# Supplementary figures and images for: Meta-Analysis of Gene Expression Changes in the Blood of Patients with Mild Cognitive Impairment and Alzheimer’s Disease Dementia
Source: Int J Mol Sci. 2019 Oct 30;20(21):5403. doi: 10.3390/ijms20215403 (PMC6862214; doi:10.3390/ijms20215403)

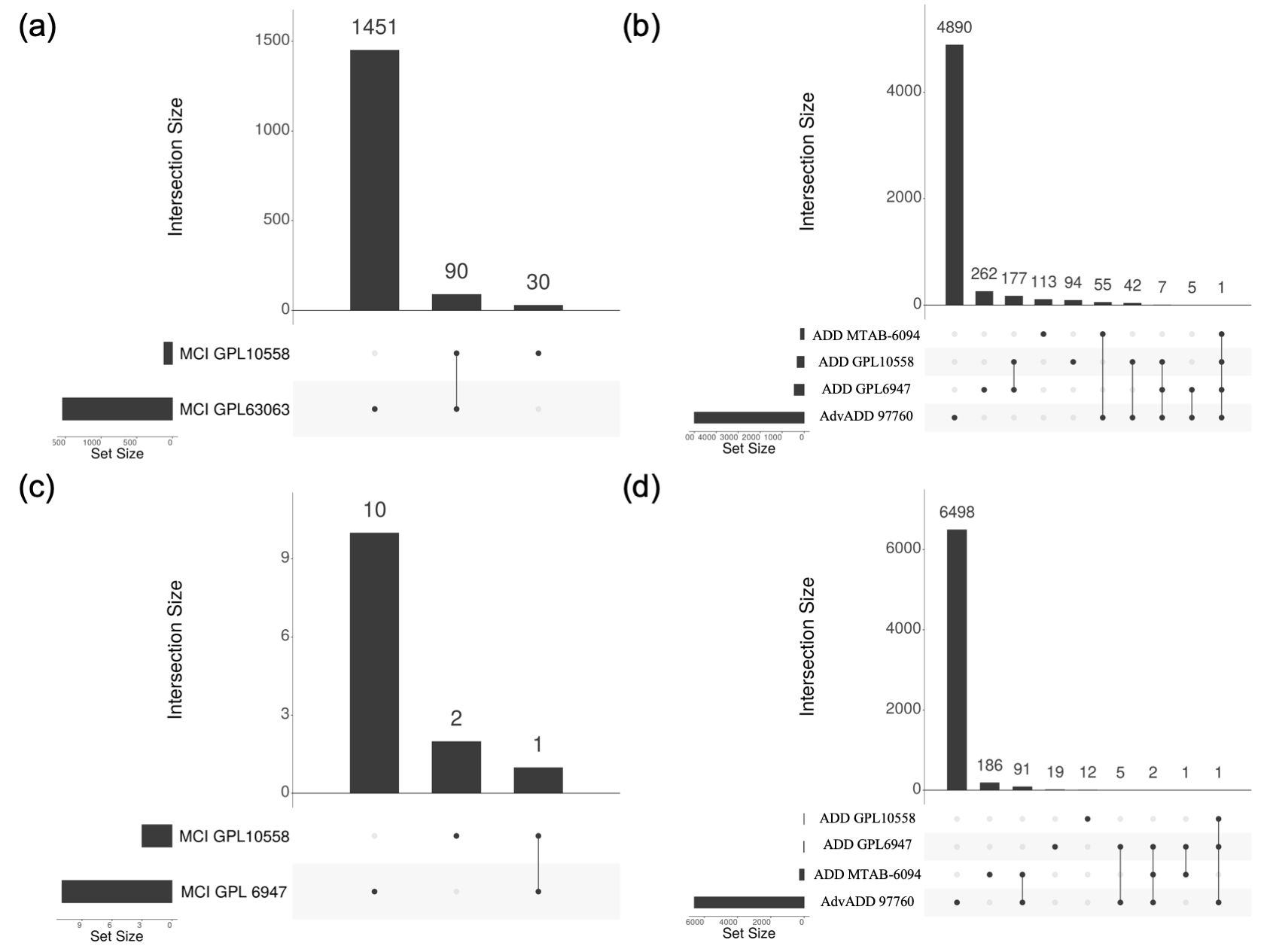

Supplement: Supplementary file 1 [file ijms-20-05403-s001.zip › Fig_S1 revised.tiff]
